# Rapidly evolving genetic features for desert adaptations in *Stipagrostis pennata*

**DOI:** 10.1186/s12864-021-08124-w

**Published:** 2021-11-23

**Authors:** Xixu Ding, Tingting Zhang, Lei Ma

**Affiliations:** College of Life Sciences, Shihezi University, Shihezi City, Xinjiang China

## Abstract

**Background:**

*Stipagrostis pennata* is distributed in the mobile and semi-mobile sand dunes which can adapt well to extreme environments such as drought and high temperature. It is a pioneer plant species with potential for stabilizing sand dunes and ecological restoration. It can settle on moving sand dunes earlier than other desert plants. It can effectively improve the stability of sand dunes and help more plants settle down and increase plant diversity. However, despite its important ecological value, the genetic resources available for this species are limited.

**Results:**

We used single-molecule real-time sequencing technology to obtain the complete full-length transcriptome of *Stipagrostis pennata*, including 90,204 unigenes with an average length of 2624 bp. In addition, the 5436 transcription factors identified in these unigenes are rich in stress resistance genes, such as MYB-related, C3H, bHLH, GRAS and HSF, *etc*., which may play a role in adapting to desert drought and strong wind stress. Intron retention events are abundant alternative splicing events. *Stipagrostis pennata* has experienced stronger positive selection, accelerating the fixation of advantageous variants. Thirty-eight genes, such as CPP/TSO1-like gene, have evolved rapidly and may play a role in material transportation, flowering and seed formation.

**Conclusions:**

The present study captures the complete full-length transcriptome of *Stipagrostis pennata* and reveals its rapid evolution. The desert adaptation in *Stipagrostis pennata* is reflected in the regulation of gene expression and the adaptability of gene function. Our findings provide a wealth of knowledge for the evolutionary adaptability of desert grass species.

**Supplementary Information:**

The online version contains supplementary material available at 10.1186/s12864-021-08124-w.

## Background

*Stipagrostis pennata* is a pioneer plant species in the desert. It grows on mobile and semi-mobile sand dunes and has excellent sand fixation ability. It can colonize mobile dunes earlier than other plants. It is reported to be diploid (2n = 2x = 22) or tetraploid (2n = 4x = 44) [1]. Compared with other desert plants, it has excellent restoration properties such as drought resistance, sand burial resistance, and wind erosion resistance. The colonization of *Stipagrostis pennata* can effectively improve the stability of sand dunes and help more plants settle and increase plant diversity.

*Stipagrostis pennata* has evolved various mechanisms to adapt to desert environment. It has a developed root system and sheath structure. It can settle in flowing sand dunes and grow well. Besides, the reproduction of *Stipagrostis pennata* is guaranteed in arid environments through various characteristics. For example, its curled leaves reduce water evaporation, the flowering time is short, and the number of seeds is large [2–5].

However, the lack of genetic information on *Stipagrostis pennata* has hinders research on its adaptation to stress. In the present study, we combined single-molecule real-time (SMRT) and second-generation sequencing (SGS) to generate the full-length *Stipagrostis pennata* transcriptome. We analyzed the gene function and structure of the *Stipagrostis pennata*, and identified alternative splicing. In order to identify the genes necessary for the adaptive evolution of *Stipagrostis pennata*, selection pressure analysis was performed. Based on the functional annotations of rapidly evolving genes, the adaptive evolution law of specific gene functions are initially revealed. The present study will provide important genetic resources for elucidating the adaptive evolution mechanism of *Stipagrostis pennata*, and will also help to better understand the evolutionary adaptability of desert grass species.

## Results

### Transcriptome sequencing

We obtained 6,128,860 subreads from raw data by removing joints and low-quality reads. The average subreads length is 2172 bp. After screening, 382,837 circular consensus sequences and 294,509 full-length non-chimeric read sequences (FLNC) were generated, with an average length of 2509 bp.

After error correction and removal of redundant transcripts, a total of 90,204 non-redundant transcripts were produced. Each represents a unique full-length transcript with an average length of 2624 bp and an N50 of 2788 bp (Additional file 1: Table S1).

### Annotation of transcripts

The 84,610 (93.80%) sequences are annotated in at least 1 of the 7 databases [6–8] (Nr, Nt, Pfam, KOG, Swiss-prot, KEGG, and GO) (Additional file 1: Figure S1). Most genes (91.28%) are annotated in the Nr database. Moreover, 33,204 unigenes are annotated in all 5 databases. Relatively small number of unigenes (347) are only annotated in the Nr database (Additional file 1: Figure S2). By searching the Nr database, 34.4%, 23.5%, 14.01% and <10% of the unigene sequences of *Stipagrostis pennata* match the corresponding sequences of *Setaria italica*, *Sorghum bicolor*, *Dichanthium oligosanthes* and *Zea mays*, respectively (Additional file 1: Figure S3).

For Gene Ontology analysis (GO), in the category of biological process (BP), the main subcategories of the classified genes include metabolic processes, cellular processes, and single-organism processes. Cell and cell parts were the most abundant terms of the cellular component (CC). In terms of the molecular function (MF) category, binding and catalytic activity are main subcategories (Additional file 1: Figure S4).

EuKaryotic Ortholog Groups (KOG) annotation showed that the most abundant genes are related to post-translational modification, protein turnover, and chaperones. This indicates that post-translational modification of proteins and regulation of protein-level modifications are very active in *Stipagrostis pennata*. The “general function prediction only” group has more abundant genes. This group mainly corresponds to conventional gene functions (Additional file 1: Figure S5).

KEGG annotation analysis was performed using unigenes. In the cellular process category, the most abundant genes are related to transport and catabolism (Additional file 1: Figure S6). In terms of environmental information processing, the most abundant genes are related to signal transduction. The number of genes related to transcription and translation is highly enriched in information processing, while the number of genes related to gene replication and repair is less. There are many genes enriched in metabolism-related pathways, and carbohydrate metabolism is dominant.

### Identification of putative transcription factors (TFs)

**Fig. 1:**
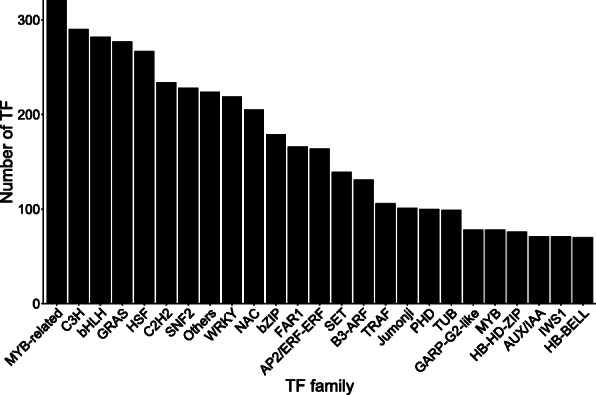
Major transcription factors identified in *Stipagrostis pennata*

**Fig. 2:**
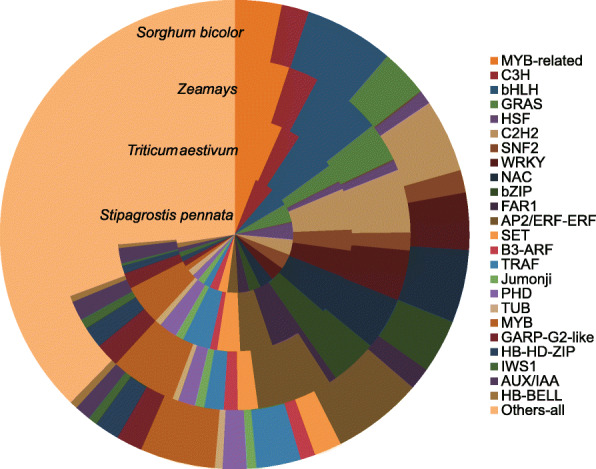
The proportion of major TFs in *Stipagrostis pennata*, *Triticum aestivum*, *Zea mays* and *Sorghum bicolor*

We identified 5436 TFs in *Stipagrostis pennata.* The most abundant families of TF are: MYB-related (321 genes), C3H (290 genes), bHLH (282 genes), GRAS (277 genes), HSF (267 genes), C2H2 (234 genes), and SNF2 (228 genes) (Fig. 1). By comparing the number of TFs in *Stipagrostis pennata* and their proportion in three species of Gramineae, it is found that the proportion of main TFs in *Stipagrostis pennata* is special (Fig. 2). The proportion of C3H, GRAS and HSF in *Stipagrostis pennata* are greater than the other three gramineous crops. These TF families play a role in stress resistance. For example, a key role of the C3H gene family is lignin synthesis [9], and the HSF gene family is known to be the key regulatory genes for heat stress adaptation [10].

### Identification of alternative splicing (AS) in *Stipagrostis pennata*

**Fig. 3:**
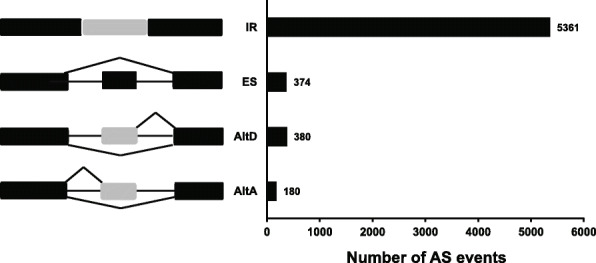
An overview of main alternative splicing events in *Stipagrostis pennata* transcripts. The left panel shows the icon of the splicing event. AltA, alternate acceptor site; AltD, alternate donor site; ES, exon skip; RI, retained intron

Intron retention (IR) are the most AS event in *Stipagrostis pennata*. It accounts for approximately 85% of the total number of AS events (Fig. 3). In addition, exon skipping (ES) and alternative donor sites (AltD) each accounts for 6% of the total number of AS events. Alternative acceptor sites (AltA) are the least AS events.

**Fig. 4:**
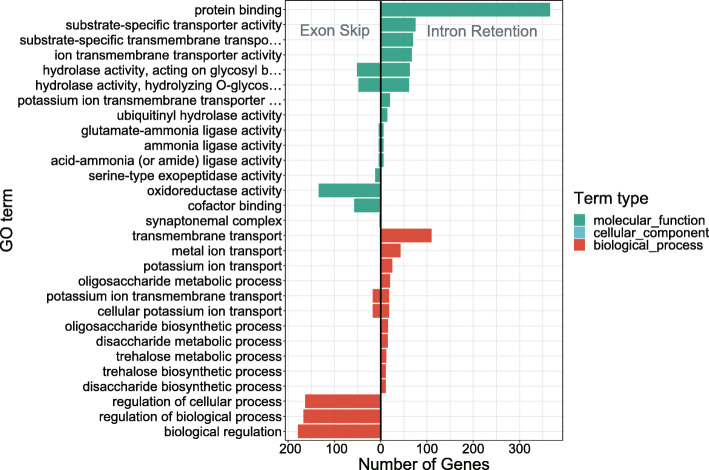
Intron retention and exon skipping are functionally different

Intron retention (IR) and exon skipping (ES) are functionally different (Fig. 4). IR events are rich in transportation-related genes. For example, there are many IR genes for protein binding and transporter activity. However, the ES genes are not annotated in the above GO terms. In contrast, ES genes are rich in oxidoreductase activity and biological process regulation. These findings may provide insight into the regulatory mechanisms that support *Stipagrostis pennata*'s adaptation to adverse environments.

### Identification of orthologous genes

**Fig. 5:**
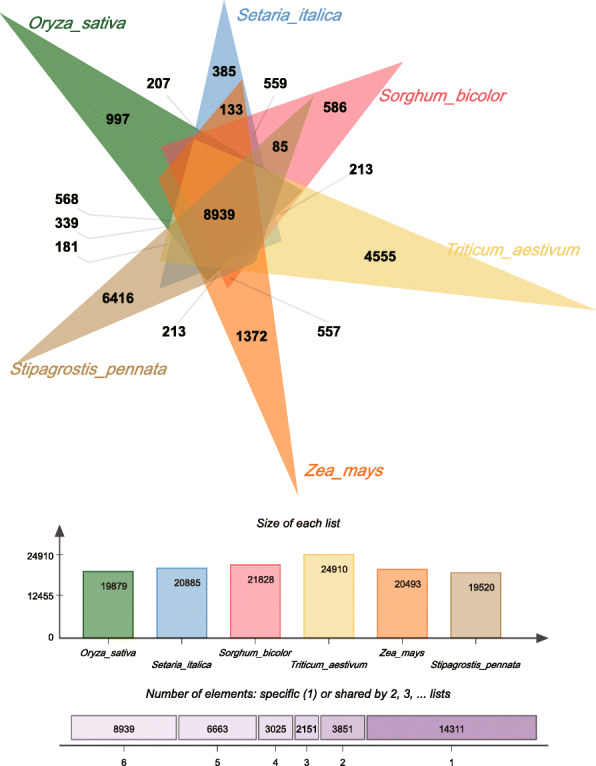
Homologous subgoups of *Stipagrostis pennata*, *Oryza sativa, Setaria italica, Triticum aestivum, Sorghum bicolor*, and *Zea mays*

Compared with other gramineous crops, *Stipagrostis pennata* gradually formed a unique desert adaptation mechanism during the evolution process. We analyzed the homology between *Stipagrostis pennata*, *Arabidopsis thaliana*, *Oryza sativa, Setaria italica, Triticum aestivum, Sorghum bicolor* and *Zea mays*. For *Stipagrostis pennata*, there are fewer genes shared (7708 sequences) with *Arabidopsis thaliana* than with the other five cereal crops. *Stipagrostis pennata* and *Sorghum bicolor* have the highest number of orthologs. The number of homologous sequences unique to a single species is 14,296. The number of orthologous genes shared among all species is 8839. There are 6416 orthologous genes found only in *Stipagrostis pennata* (Fig. 5).

**Fig. 6:**
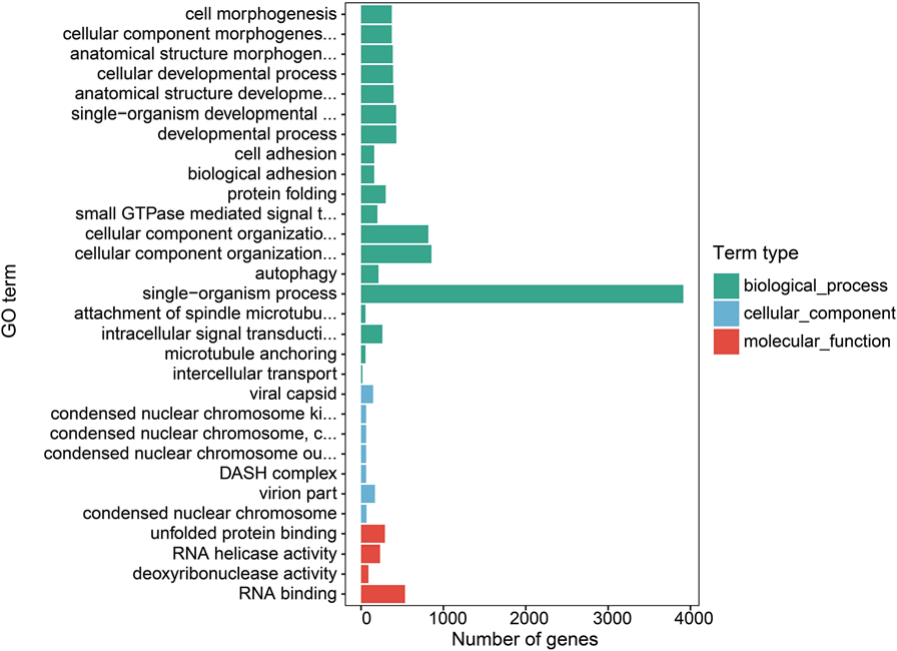
Enrichment analysis of *Stipagrostis pennata-specific genes* using hypergeometric test (*p*-value < 0.05)

**Fig. 7:**
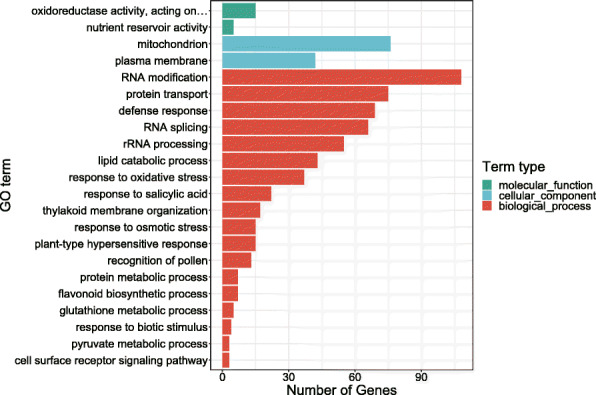
Enrichment analysis of orthologous genes using hypergeometric test (*p*-value < 0.05)

In order to analyze the composition and function of the orthologous genes shared by above-mentioned gramineous plants, GO function enrichment was performed on orthologous genes. *Stipagrostis pennata*'s unique genes are mainly enriched in the single biological process (Fig. 6). The 8839 orthologous genes shared by all species were significantly enriched to 22 GO entries. Four of those entries belong to the categories of molecular functions and cellular components. The remaining entries belong to the category of biological processes. This result indicates that the orthologous genes are mainly involved in biological processes (Fig. 7).

In the category of biological processes, most orthologous genes are related to RNA modification. RNA modification is important for regulating of RNA biological functions. In addition, there are many genes related to RNA splicing, RNA shearing and rRNA processing. Furthermore, orthologous genes are also related to protein transport, defense response, lipid catabolism, oxidative stress and osmotic stress. In the category of cellular components, orthologous genes are related to mitochondrion and plasma membrane. Finally, in the molecular function category, the orthologous genes are mainly related to oxidoreductase activity and nutrient reservoirs.

### Selection pressure analysis

**Fig. 8:**
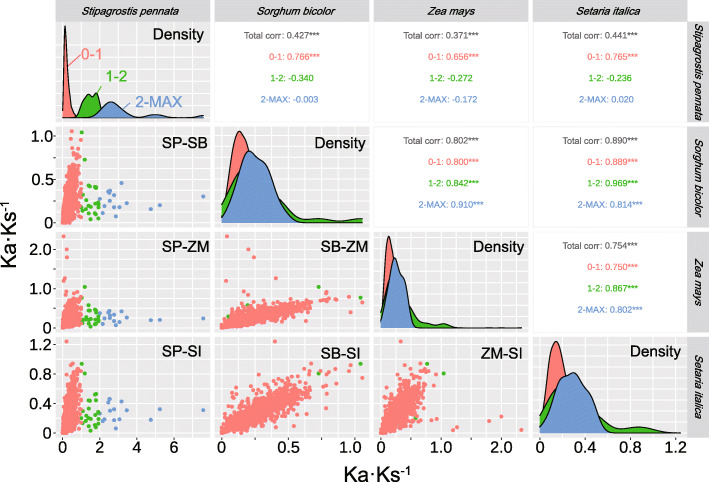
Distribution and correlation of ω (Ka·Ks^− 1^) values. The ω values are calculated in the gene pairs of the target species and* Oryza sativa*. The subset on the diagonal shows the ω density. Genes are grouped according to *Stipagrostis pennata* ω bins (0 ~ 1, 1 ~ 2, 2 ~ maximum) and coloured with red, green, and blue. The subset in the lower left triangle shows a scatter plot of gene ω between two species and the symmetrical one in the lower left shows the Spearman coefficient of the entire area and three bins

*Stipagrostis pennata* is a pioneer Gramineae and can adapt to harsh desert environments. An in-depth understanding of the evolutionary pressures of *Stipagrostis pennata* can provide insight for subsequent genetic mining. To gain further insight, *Oryza sativa* was selected as a reference species to analyze the selection pressure of orthologous genes of *Stipagrostis pennata*, *Sorghum bicolor*, *Zea mays*, *Triticum aestivum*, and *Setaria italica*. The ratio of non-synonymous substitutions to synonymous substitutions ω (Ka·Ks^− 1^) values were calculated in the gene pairs of the target species and *Oryza sativa*. Figure 8 shows that when the ω value is greater than 1, the ω value distribution of *Stipagrostis pennata* is different from the other four crops. High ω is a typical feature of rapid evolution. This indicates that *Stipagrostis pennata* has undergone species-specific genetic variation while adapting to the harsh desert environment.

As the value of ω increases, the difference between *Stipagrostis pennata* and the other four grasses become more obvious. According to the ω values of *Stipagrostis pennata*, homologous gene pairs were divided into three groups. These groups were: 0 ~ 1, 1~ 2, and 2 ~ max of the value of ω. The Spearman rank correlation coefficients of ω were calculated in those groups, respectively. As shown in Fig. 8, in the total area of ω, all four species are highly significantly positively correlated with each other (*P* < 0.01). However, when the ω value is greater than 1, the correlation between *Stipagrostis pennata* and  *Sorghum bicolor*, *Zea mays*, *Triticum aestivum* and *Setaria italica* is not significant. This indicates that positive selection in *Stipagrostis pennata* is dfferent to the other grasses. These genes with increased molecular evolutionary rate (ω) may be the key to understanding the adaptation of *Stipagrostis pennata* to the desert.

**Fig. 9:**
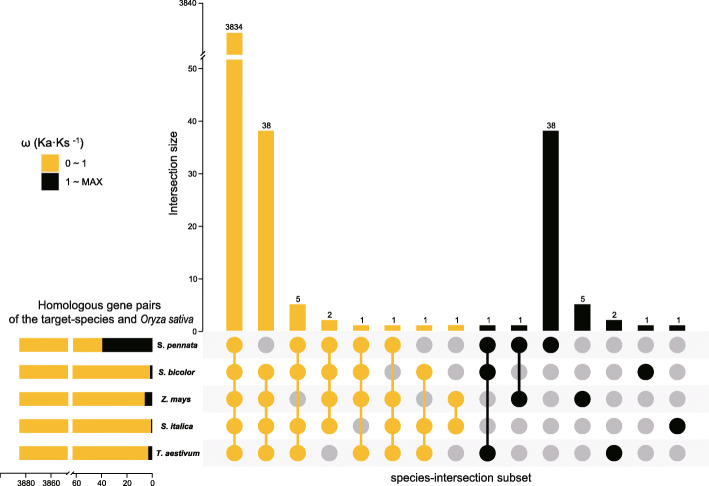
Intersections of the homologous genes of *Stipagrostis pennata*, *Sorghum bicolor*, *Zea mays*, *Setaria italica* and *Triticum aestivum*. Genes are divided into two groups according to their ω (Ka·Ks^− 1^) values: 0 ~ 1 (yellow) and 1 ~ max (black). These values are calculated in the gene pairs of the target species and *Oryza sativa*

Although most genes are shared among all plants and are under purification selection, there are 38 Stipagrostis pennata-specific genes under positive selection (Fig. 9, Additional file 2). Some of these species-specific genes are related to transporter activity in the category of molecular function. They are involved in ion binding, heterocyclic compound binding, organic cyclic compound binding and protein binding. Furthermore, some genes are related to post-transcriptional modification and protein modification.

**Fig. 10:**
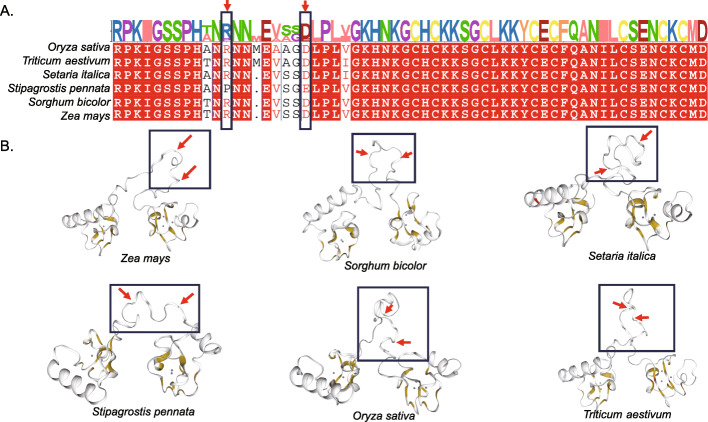
*CPP/TSO1-like* homologous gene in six Gramineae species. (**A**) Amino acid sequence alignment and variation site. The arrow indicates the amino acid substitution site. (**B**) Three dimensional structure of CPP/TSO1-like protein. The three-dimensional structures are predicted using SWISS-MODEL. The box shows the CXC motif-linked region

Rapidly evolving genes may play a role in the desert adaption process. For example, the gene i2_LQ_SP_c5507/f1p1/2479 is a rapidly evolving gene of *Stipagrostis pennata*. This gene is a *CPP/TSO1-like* gene with two cysteine-rich CXC motifs. It plays a key role in plant reproductive tissue formation (including plant flower formation). We compared this gene with the genes of five other Gramineae species. In the CXC motif region, the sequences of the six species were generally consistent. However, in the region where the two CXC motifs join, *Stipagrostis pennata* has some differences in amino acid positions. As shown in Fig. 10A, in *Stipagrostis pennata*, arginine is replaced by proline and aspartic acid is replaced by glutamic acid. Due to the variation of these two loci, the structure of *Stipagrostis pennata* CPP/TSO1-like protein may be affected. Compared with the other 5 species, the three-dimensional structure of the Stipagrostis pennata protein is looser in the junction area between the CXC motifs. We hypothesize that the variant three-dimensional structure of *Stipagrostis pennata* CPP/TSO1-like protein plays a role in its adaptation to flowering and seed formation.

## Discussion

Plants living in harsh environments have undergone significant adaptations during their long evolutionary history. Research on adaptive evolution can not only have a basic understanding of the evolutionary history of this plant, but also lay the foundation for genetic mining of features related to stress resistance [11–15]. *Stipagrostis pennata* is a pioneer plant that grows in the desert. It has formed a variety of resistance mechanisms, such as drought tolerance. However, the genetic adaptation mechanism of *Stipagrostis pennata* has not yet been elucidated. In the present study, we used PacBio SMRT technology and Illumina short-read sequencing to obtain the most complete full-length transcript of *Stipagrostis pennata* to date. These findings laid the foundation for future research on *Stipagrostis pennata*.

Plant adaptation is the result of a combination of gene expression regulation and function adaptive evolution [16, 17]. Gene expression regulation helps to improve the ability of plants to adapt to changes in the external environment [18–20]. Desert environments are harsh and varied. Any plant that survives in desert environments faces complex environmental stresses. Dynamic regulation of specific gene expression and biochemical processes is particularly important. In such dynamic process, transcription factors are one of the important regulatory components [21].

The 24 TFs, such as MYB-related, C3H, bHLH, and HSF genes, are abundant transcription factors in *Stipagrostis pennata*. These transcription factor families are closely related to abiotic stress responses [22–24]. *Stipagrostis pennata*, as a pioneer plant living in mobile dunes, has tough stem tissue that can withstand strong desert winds. This tissue may be the result of changes in gene expression related to lignin and cellulose synthesis [25]. The C3H is an important regulatory gene in lignin synthesis. It is the main transcription factor that regulates the formation of plant secondary walls [26, 27]. We propose that *Stipagrostis pennata* can improve wind erosion resistance by regulating the synthesis of cellulose and lignin. Interestingly, bHLH is an important transcription factor that can regulate drought resistance of plants such as *Arabidopsis thaliana* and *Triticum aestivum.* In addition, HSF is involved in the adaptation of plants to heat stress [28–32]. Furthermore, alternative splicing is another important mechanism for regulating plant gene expression. Intron retention events are abundant alternative splicing events in *Stipagrostis pennata*. Intron retention helps diversify plant gene functions, thereby enhancing the ability of plants to adapt to the environment [33]. In *Stipagrostis pennata*, intron retention events may improve stress tolerance through flexible transcript splicing. 

The adaptive evolution of gene expression is an important mechanism for plants to adapt to changes in the external environment. However, the range of gene expression is limited. Therefore, the adaptability of gene function has a greater impact in the evolution process [34]. The homologous genes, identified in *Stipagrostis pennata* and major cereal crops, are mainly enriched in GO terms for RNA modification and processing. *Stipagrostis pennata* has species-specific genes under positive selection pressure. The 38 rapidly evolving genes unique to *Stipagrostis pennata*  may be involved in molecular translocation, binding and lo﻿calization. In addition, the molecular functions, ion binding, heterocyclic compound binding, organic cyclic compound binding and protein binding of *Stipagrostis pennata* are all positively selected.

CPP/TSO1-like genes are involved in plant flowering and seed formation. In *Arabidopsis thaliana*, mutations in this gene lead to changes in flower structure and reduced pollen levels, and affect the development of ovules and carpels. The 3-D structure shows that the junction region between the two CXC motifs of the gene in *Stipagrostis pennata* is looser due to the variation of the two sites. This variant 3D structure may help *Stipagrostis pennata* adapt to the harsh desert environment.

## Conclusions

*Stipagrostis pennata* grows in the desert and can adapt to harsh and extreme environments. It has a unique mechanism in the regulation of gene expression and the adaptive evolution of gene function. Most of its TFs is related to plant resistance regulation. IR events are main AS events in *Stipagrostis pennata*. The rapidly evolving genes may be related to molecular transport, flowering and seed formation. Finally, our findings may lay the foundation for future research on *Stipagrostis pennata*.

## Methods

### Plant materials

Fresh *Stipagrostis pennata* tissues were collected from the southern margin of the Gurbantunggut Desert in Xinjiang in China. Botanist Ping Yan from Shihezi University identified the *Stipagrostis pennata*, and the voucher specimens (No. 6704) have been deposited in the the College of Life Sciences, Shihezi University China. In order to obtain more complete transcriptome information, we collected the roots, stems, leaves, flowers, and seedlings of *Stipagrostis pennata*. The research on the *Stipagrostis pennata* complies with the IUCN Policy Statement on Research Involving Species at Risk of Extinction and the Convention on the Trade in Endangered Species of Wild Fauna and Flora. To obtain as many expressed genes as possible, five different tissues (including leaves, stems, flowers, roots, and root sheaths) were sampled. Tissue samples were frozen in liquid nitrogen and stored at − 80°C until RNA extraction.

*Arabidopsis thaliana, Triticum aestivum*, *Oryza sativa*, *Setaria italica*, *Sorghum bicolor* and *Zea mays* genomes were downloaded from the Ensembl Plants database.

### PacBio Iso-Seq library preparation

Total RNA were extracted from the samples. RNA degradation and contamination were analyzed using 1% agarose gel electrophoresis. RNA purity was measured using a Nanodrop ND-1000 spectrophotometer (Nano Drop Technologies, Wilmington, DE, USA). Precise quantification of RNA concentrations was carried out using a Qubit 2.0 Flurometer (Life Technologies, Carlsbad, CA, USA). RNA integrity was measured using Agilent 2100 technology (Agilent Technologies, Palo Alto, CA, USA).

The Iso-Seq library was prepared according to the Isoform Sequencing protocol (Iso-Seq) using the Clontech SMARTer PCR cDNA Synthesis Kit. The BluePippin Size Selection System protocol was performed as described by Pacific Biosciences (PN 100-092-800-03). Libraries were subsequently sequenced using a Pacific Biosciences sequencing machine.

### Illumina cDNA library preparation

A total of 1.5 µg RNA per sample was used for cDNA library preparation. Sequencing libraries were generated using a NEBNext® Ultra™ RNA Library Prep Kit for Illumina® according to the manufacturer’s recommendations. Index codes were added to attribute identification sequences to each sample. Library preparations were sequenced on an Illumina HiSeq 2500 platform (Illumina, San Diego, CA, USA). Sequencing was performed in a paired-end manner. All sequencing was performed in a high-throughput manner at the Novogene Bioinformatics Institute (Novogene, Beijing, China).

### Functional annotation and selection pressure analysis

The Blast2GO program [35] (http://www.blast2go.com) was used to annotate GO terms (http://www.geneontology.org) based on the NR annotations. A cutoff E-value of ≤ 1e-6 was employed. All enrichment analyses were performed using hypergeometric test (*p*-value < 0.05). Plant transcription factors were predicted using iTAK [36] software and TFDB 2.0 database [37]. The transcription factor annotations for *Triticum aestivum*, *Zea mays* and *Sorghum bicolor* were downloaded from iTAK database. We used CNCI [38], CPC [39], Pfam-scan [40], and PLEK [41] to predict coding sequences [42]. AStrap [43] was used to identify alternative splicing events in *Stipagrostis pennata*. Homologous genes were screened using OrthoVene2 [44]. Synonymous substitutions (Ka) and non-synonymous substitutions (Ks) were calculated using KaKs_calculator 2.0 [45].

## Supplementary information

**Additional file 1**

**Additional file 2**

## Data Availability

The RNA sequencing data used in this study have been uploaded to the National Center for Biotechnology Information (NCBI) as Bioproject ID: PRJNA763703.

## Abbreviations

AS: Alternative splicing
BP: Biological process
CC: Cellular component
ES: Exon skiping
FLNC: Full-length non-chimeric read sequences
GO: Gene Ontology
IR: Intron retention
Ka: The number of nonsynonymous substitutions per non-synonymous site
KEGG: Kyoto Encyclopedia of Genes and Genomes
KOG: EuKaryotic Ortholog Groups
Ks: The number of synonymous substitutions per synonymous site
MF: Molecular function
NR: NCBI non-redundant protein
NT: NCBI nucleotide sequences
Pfam: Protein family
SGS: Second-generation sequencing
SMRT: Single-molecule real-time
TF: Transcription factor
TGS: Third-generation sequencing
