# Supplementary material for: Rapidly evolving genetic features for desert adaptations in Stipagrostis pennata
Source: BMC Genomics. 2021 Nov 23;22:846. doi: 10.1186/s12864-021-08124-w (PMC8609760; doi:10.1186/s12864-021-08124-w)
Supplement: Supplementary file 1 — Additional file 1 [file 12864_2021_8124_MOESM1_ESM.docx]

**Additional file 1**

**Figures**

**Figure S1. Number of annotation results for each of the seven databases**


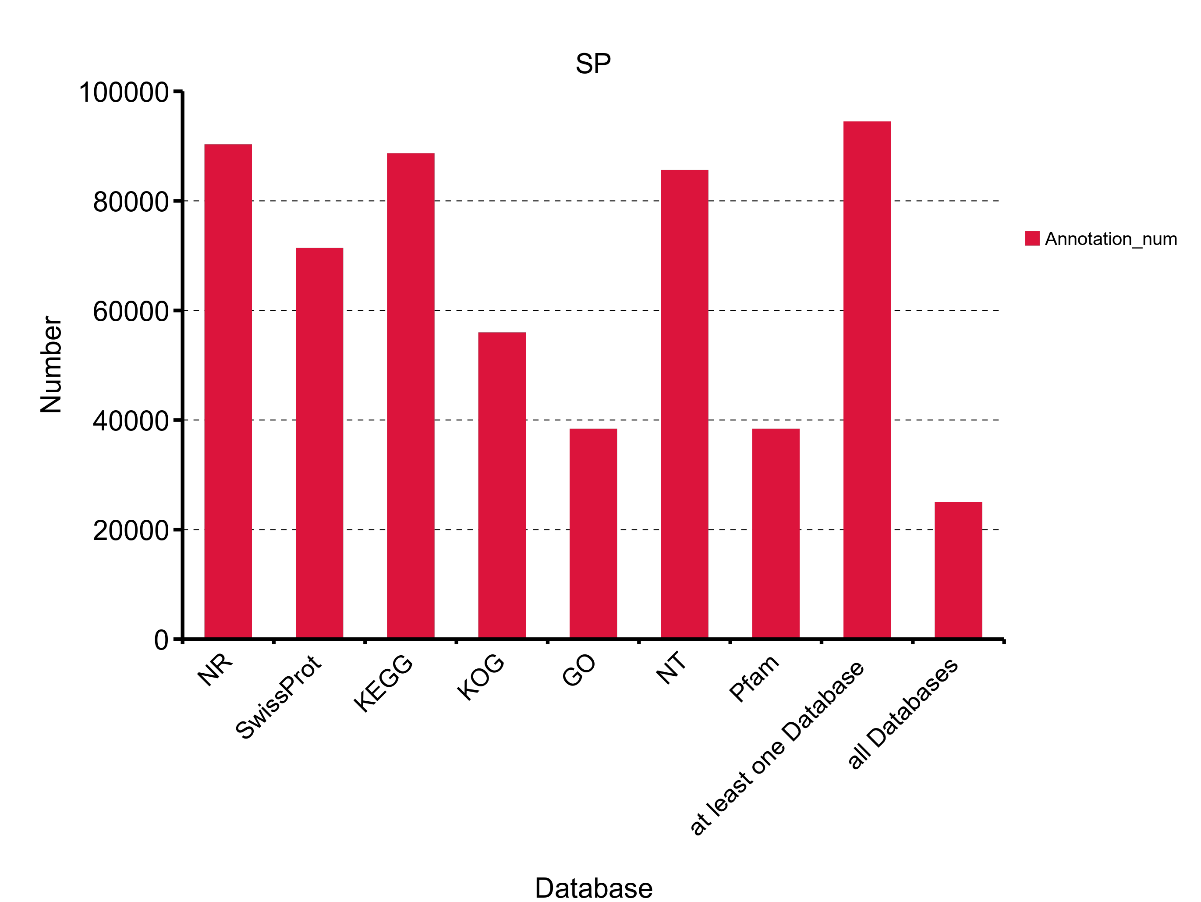


**Figure S2. Annotated Venn diagram of gene function**


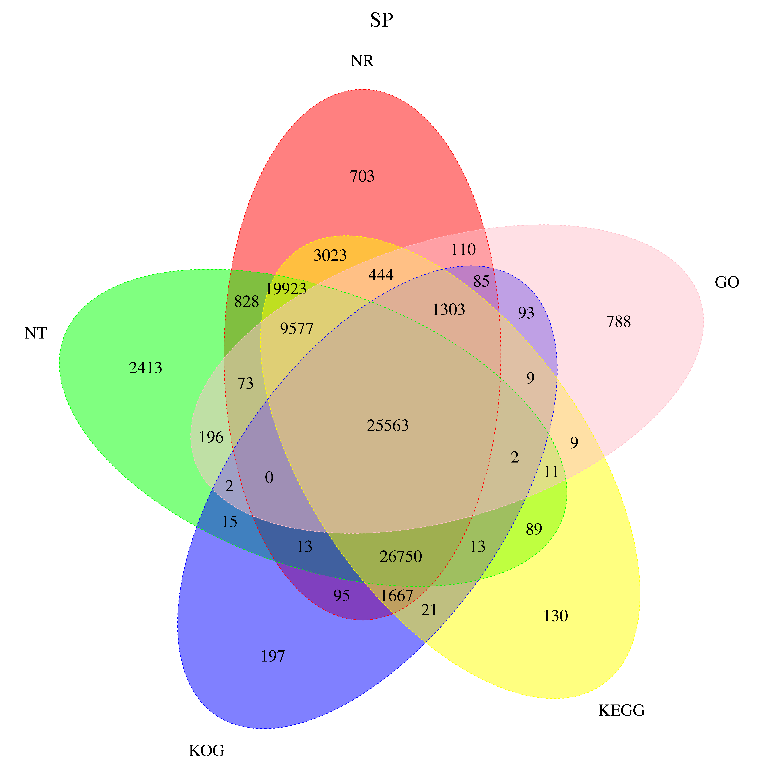


**Figure S3. Annotated matched genes for each species in the Nr database**


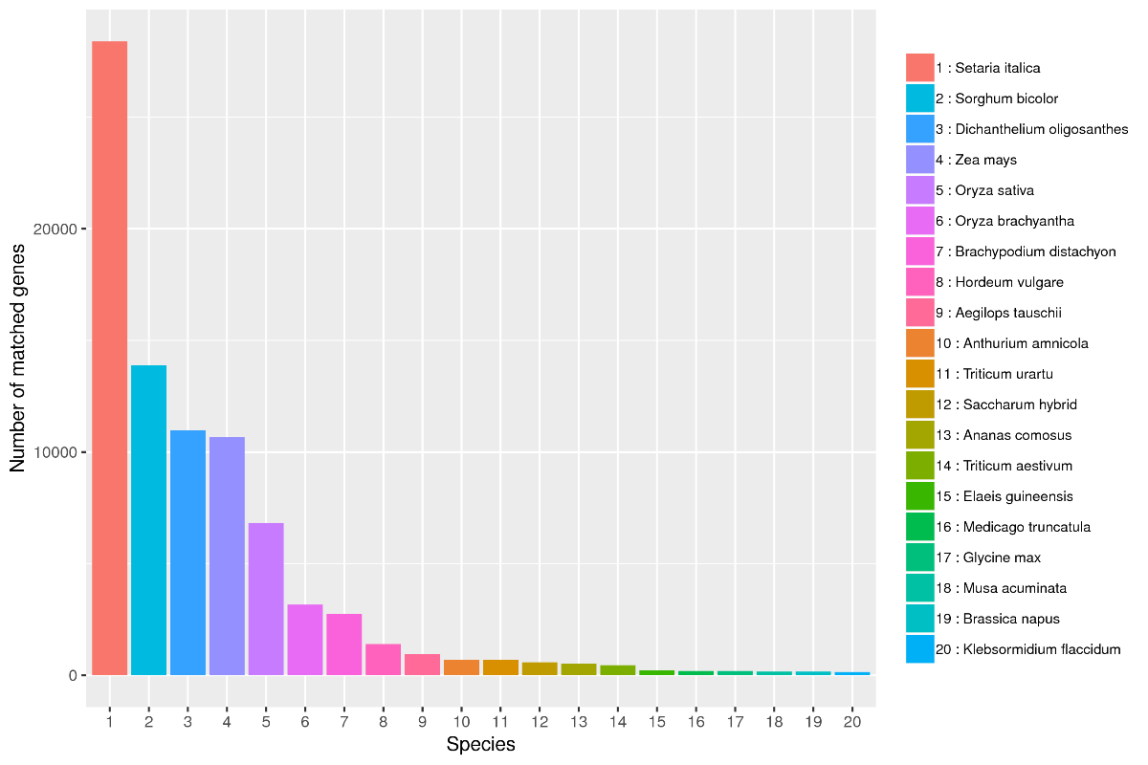


**Figure S4. Gene ontology classification of *Stipagrostis pennata* unigenes**

Enrichment analyses were performed using a hypergeometric test (*p*-value < 0.05).


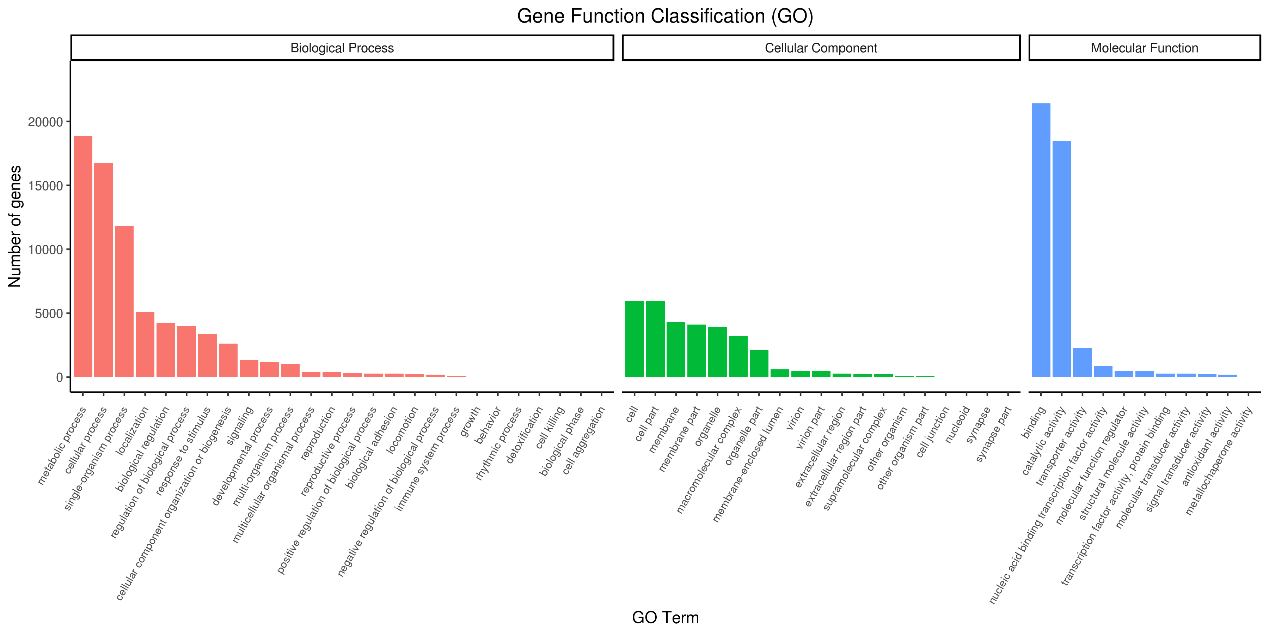


**Figure S5. KOG annotation results**


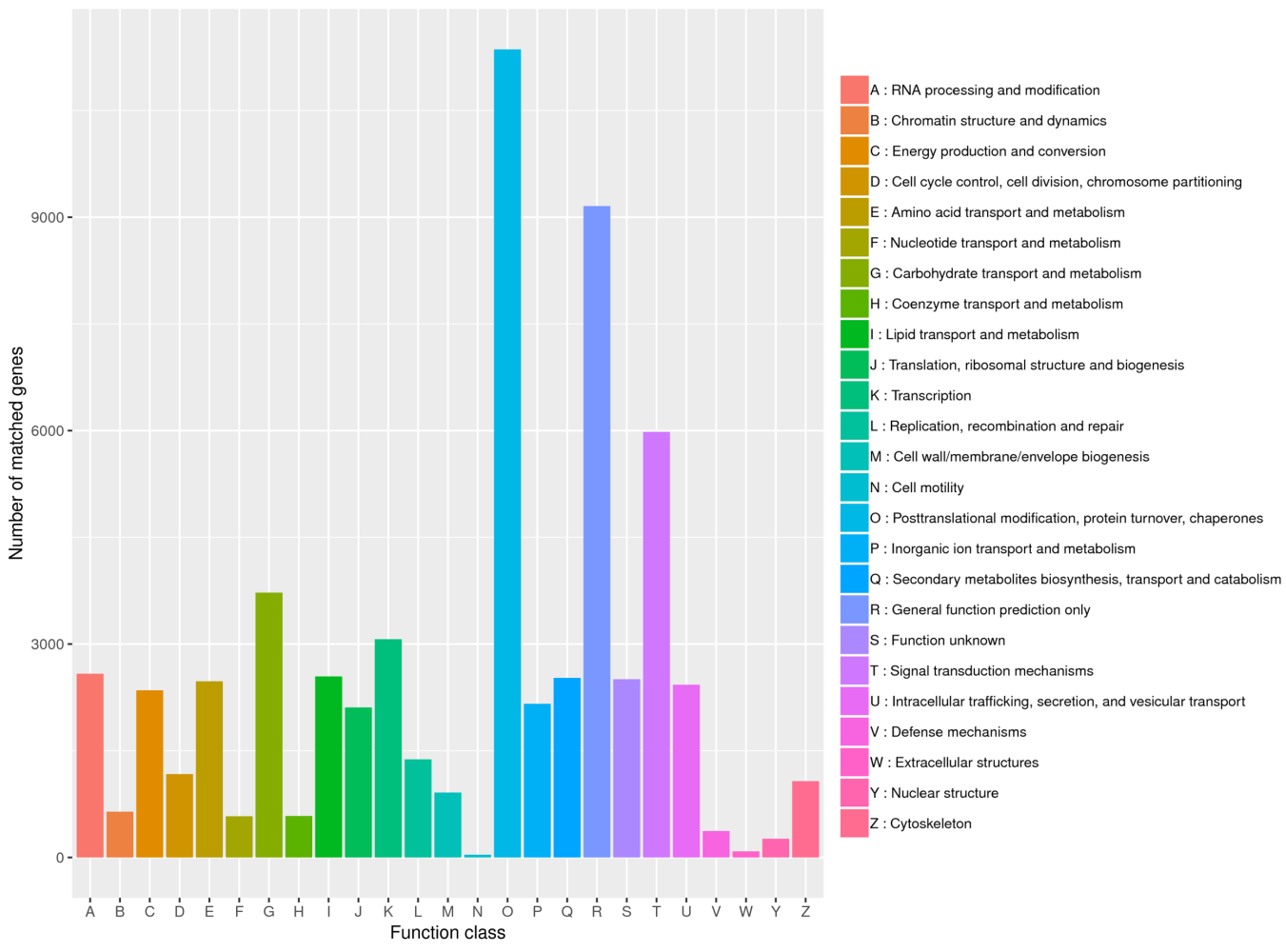


**Figure S6. KEGG annotation results**

Enrichment analyses were performed using a hypergeometric test (*p*-value < 0.05).


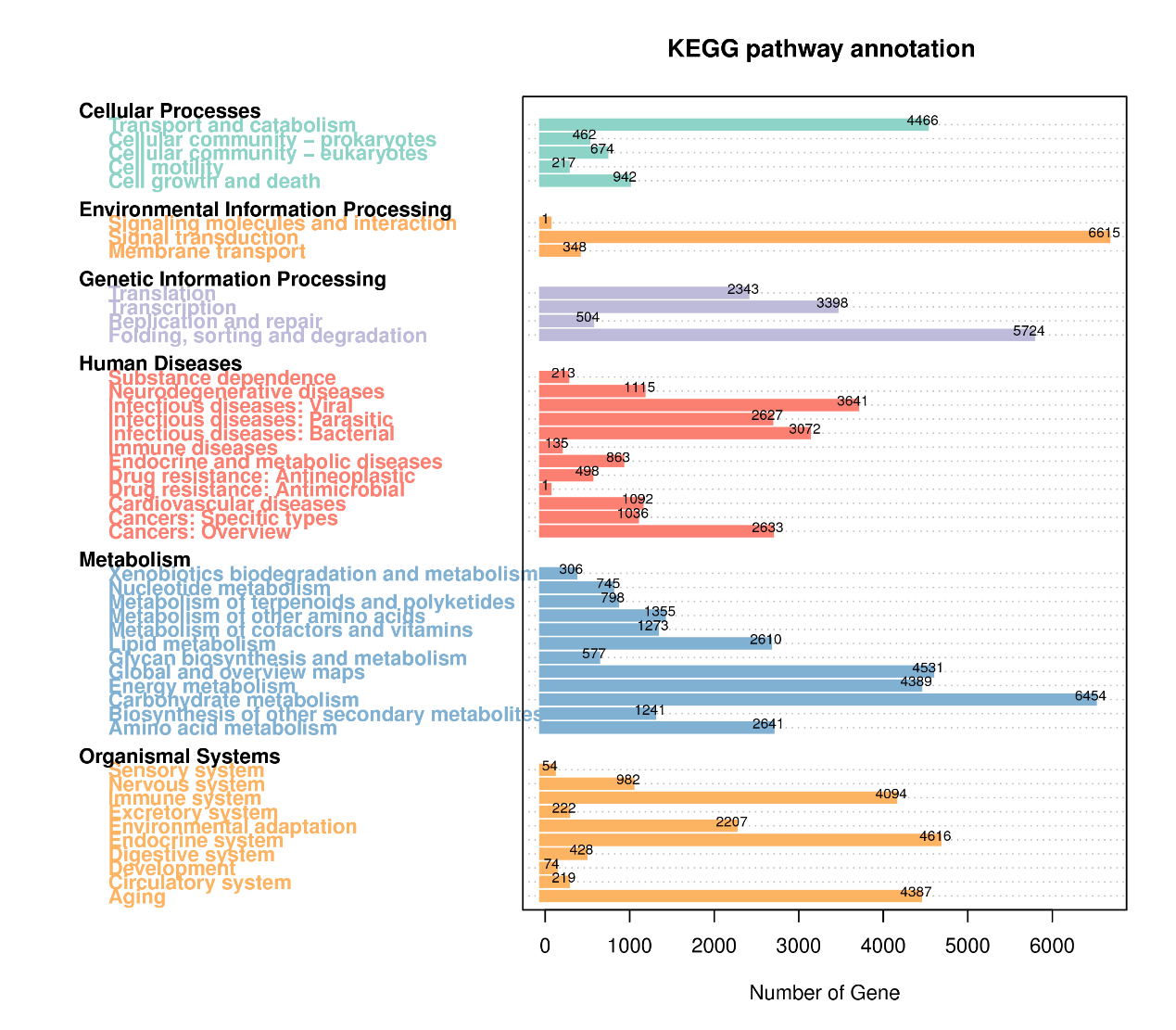


**Figure S7**. **The distribution of Ka·Ks-1 values on homologous genes in six plant species with *Oryza sativa* as the outgroup species**


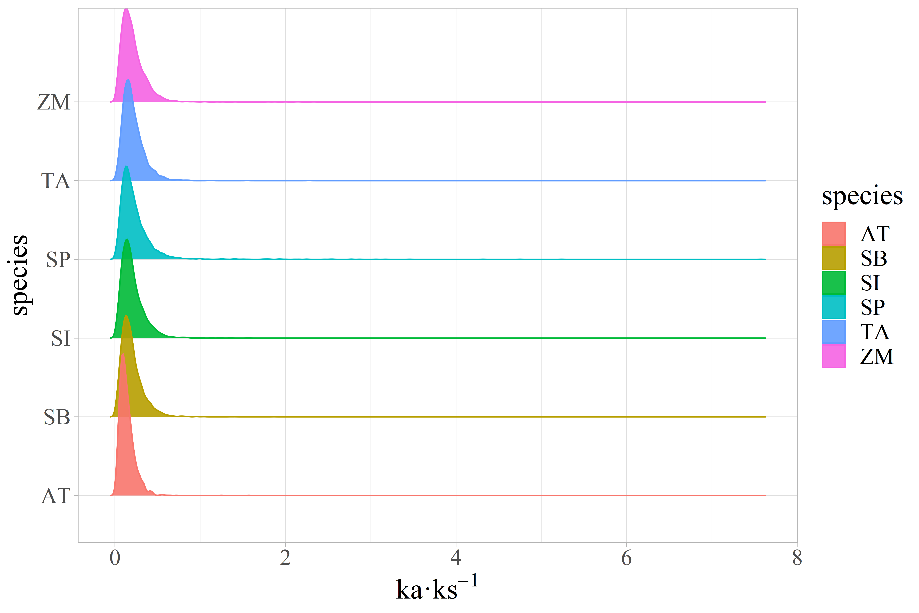


**Tables**

**Table S1**. ***Stipagrostis pennata* transcript lengths before and after correction**

| Type | Total_nucleotides | Total_number | Mean_length | Min_length | Max_length | N50 | N90 |
| --- | --- | --- | --- | --- | --- | --- | --- |
| Before  correct | 415,325,627 | 158,689 | 2,618 | 177 | 14,484 | 2,783 | 1,875 |
| After  correct | 416,380,982 | 158,689 | 2,624 | 176 | 14,445 | 2,788 | 1,879 |
